# The importance of Indigenous Peoples’ lands for the conservation of terrestrial mammals

**DOI:** 10.1111/cobi.13620

**Published:** 2020-12-30

**Authors:** Christopher J. O'Bryan, Stephen T. Garnett, Julia E. Fa, Ian Leiper, Jose A. Rehbein, Álvaro Fernández‐Llamazares, Micha V. Jackson, Harry D. Jonas, Eduardo S. Brondizio, Neil D. Burgess, Catherine J. Robinson, Kerstin K. Zander, Zsolt Molnár, Oscar Venter, James E. M. Watson

**Affiliations:** ^1^ School of Earth and Environmental Sciences The University of Queensland Brisbane QLD 4072 Australia; ^2^ Centre for Biodiversity and Conservation Science The University of Queensland Brisbane QLD 4072 Australia; ^3^ Research Institute for the Environment and Livelihoods Charles Darwin University Darwin NT 0909 Australia; ^4^ Division of Biology and Conservation Ecology School of Science and the Environment Manchester Metropolitan University Manchester M15 5RN U.K.; ^5^ Center for International Forestry Research Situ Gede Bogor 16115 Indonesia; ^6^ Environment, Natural Resources, & the Blue Economy Global Practice The World Bank Washington DC 20433 U.S.A.; ^7^ Helsinki Institute of Sustainability Science (HELSUS) University of Helsinki Helsinki 00014 Finland; ^8^ Future Law Kota Kinabalu Sabah 88300 Malaysia; ^9^ Department of Anthropology Indiana University Bloomington IN 46202 U.S.A.; ^10^ Center for Macroecology Evolution and Climate University of Copenhagen Copenhagen DK‐2100 Denmark; ^11^ United Nations Environment Programme World Conservation Monitoring Center (UNEP‐WCMC) Cambridge CB3 0DL U.K.; ^12^ Commonwealth Science & Industrial Research Organisation (CSIRO) Brisbane QLD 4102 Australia; ^13^ Northern Institute Charles Darwin University Darwin NT 0909 Australia; ^14^ Centre for Ecological Research Institute of Ecology and Botany Vácrátót 2163 Hungary; ^15^ Natural Resource and Environmental Studies Institute University of Northern British Columbia 3333 University Way Prince George BC V2N 4Z9 Canada; ^16^ Global Conservation Program Wildlife Conservation Society 2300 Southern Boulevard Bronx NY 10460 U.S.A.

**Keywords:** AOH, area of habitat, biodiversity conservation, distributions, endangered species, landscape conservation, sustainable development goals, traditional owners, área del hábitat, ADH, conservación de la biodiversidad, conservación del paisaje, distribuciones, dueños tradicionales, especies en peligro, metas de desarrollo sustentable, 生物多样性保护, 栖息地范围 (AOH), 物种分布, 濒危物种, 景观保护, 可持续发展目标, 传统所有者

## Abstract

Indigenous Peoples’ lands cover over one‐quarter of Earth's surface, a significant proportion of which is still free from industrial‐level human impacts. As a result, Indigenous Peoples and their lands are crucial for the long‐term persistence of Earth's biodiversity and ecosystem services. Yet, information on species composition on these lands globally remains largely unknown. We conducted the first comprehensive analysis of terrestrial mammal composition across mapped Indigenous lands based on data on area of habitat (AOH) for 4460 mammal species assessed by the International Union for Conservation of Nature. We overlaid each species’ AOH on a current map of Indigenous lands and found that 2695 species (60% of assessed mammals) had ≥10% of their ranges on Indigenous Peoples’ lands and 1009 species (23%) had >50% of their ranges on these lands. For threatened species, 473 (47%) occurred on Indigenous lands with 26% having >50% of their habitat on these lands. We also found that 935 mammal species (131 categorized as threatened) had ≥ 10% of their range on Indigenous Peoples’ lands that had low human pressure. Our results show how important Indigenous Peoples’ lands are to the successful implementation of conservation and sustainable development agendas worldwide.

## Abstract 2

*Article impact statement*: Indigenous Peoples’ lands are important for the successful implementation of international conservation and sustainable development agendas.

## Abstract (es)

La Importancia de las Tierras de los Pueblos Indígenas para la Conservación de los Mamíferos Terrestres

**Resumen**

Las tierras pertenecientes a pueblos indígenas cubren más de un cuarto de la superficie del planeta, una proporción importante que se encuentra aún libre de impactos humanos a nivel industrial. Como resultado, los pueblos indígenas y sus tierras son cruciales para la persistencia a largo plazo de la biodiversidad en la Tierra y de los servicios ecosistemicos. Sin embargo, la información sobre la composición de especies en estas tierras a nivel mundial todavía permanece desconocida en su mayoría. Realizamos el primer análisis integral de la composición de mamíferos terrestres a lo largo de las tierras indígenas mapeadas con base en los datos sobre el área del hábitat (ADH) de 4,460 especies de mamíferos valorados por la Unión Internacional para la Conservación de la Naturaleza. Sobrepusimos el ADH de cada especie en un mapa actual de tierras indígenas y encontramos que 2,695 especies (60% de los mamíferos valorados) tienen ≥10% de su distribución dentro de tierras de pueblos indígenas y que 1,009 especies (23%) tienen >50% de su distribución dentro de estas tierras. De las especies amenazadas, 473 (47%) ocurrieron en tierras indígenas.También descubrimos que 935 especies de mamíferos (131 categorizadas como amenazadas) tienen ≥ 10% de su distribución dentro de tierras de pueblos indígenas con baja presión humana. Nuestros resultados muestran cuán importantes son las tierras de los pueblos indígenas para la implementación exitosa de la conservación y las agendas globales de desarrollo sustentable.

## 【摘要】

原住民的土地占地球表面四分之一以上的面积, 其中有很大一部分尚未受到人类工业的影响。因此, 原住民及其土地对地球生物多样性和生态系统服务的长期续存至关重要。然而, 在全球范围内, 关于这些土地上物种组成的信息仍然很有限。我们根据国际自然保护联盟评估的 4460 种哺乳动物栖息地的数据, 首次对原住民土地上陆生哺乳动物的组成进行了全面分析。我们将每个物种的栖息地范围叠加在现有的原住民土地地图上, 发现 2695 个物种 (占已评估哺乳动物的 60%) 有 10% 以上的分布范围与原住民土地重合, 1009 个物种 (占 23%) 在原住民土地上的分布面积超过其栖息地面积的 50% 。受威胁物种中有 473 种 (占 47%) 在原住民土地上有分布。我们还发现, 935 种哺乳动物 (其中 131 种被列为濒危物种) 有超过 10% 的栖息地位于人类压力较低的原住民土地上。我们的研究结果强调了原住民土地对于全球成功实施保护和可持续发展议程的重要性。**【翻译:胡怡思;审校:聂永刚】**

## Introduction

Through well‐established traditional knowledge systems and governance practices, Indigenous Peoples are the environmental stewards of their lands. This is gradually being recognized in domestic and international policy (IPBES 2019). Indigenous Peoples’ lands cover at least one‐quarter of terrestrial Earth and overlap with 37% of all terrestrial protected areas and 40% of landscapes without industrial‐level human impacts (Garnett et al. 2018). Some countrywide assessments demonstrate the importance of Indigenous Peoples’ lands in terms of the biodiversity contained in them. In Australia, for example, 45–60% of the country's threatened species occur on Indigenous Peoples’ lands (Renwick et al. 2017; Leiper et al. 2018) and vertebrate biodiversity is equal in Indigenous Peoples' lands and protected areas in 3 countries (Australia, Brazil, and Canada; Schuster et al. 2019). However, global assessments of the overlap between Indigenous Peoples’ lands, including areas free from industrial‐level human impacts, and species distributions (including threatened species) are lacking. Regions free from industrial‐level human impacts are likely to be of high conservation value (Di Marco et al. 2018), given the connection between land‐use transformation and species declines (Newbold et al. 2015; Tilman et al. 2017). These landscapes may also be important ecological refugia (Scheffers et al. 2016; Allan et al. 2019), offering some protection against the pressures of expanding resource extraction frontiers (Rehbein et al. 2020).

We conducted to our knowledge the first global assessment of the overlap between mapped Indigenous Peoples’ lands (Garnett et al. 2018) and mapped terrestrial mammal area of habitat (AOH) (Rondinini et al. 2011). We also assessed mammal species composition on low‐pressure Indigenous Peoples’ lands based on human footprint data (Williams et al. 2020). These results are relevant to the development and implementation of the post‐2020 Global Biodiversity Framework agreement that will emerge from the Convention on Biological Diversity's (CBD) discussions on abating species extinctions and reducing the erosion of ecosystem services (CBD 2018), as well as for countries trying to implement actions to achieve the 2030 United Nation's Sustainable Development Goals.

## Methods

### Spatial Data on Species Area of Habitat and Indigenous Peoples’ Lands

We focused our analysis on terrestrial mammals that have been comprehensively assessed by the International Union for Conservation of Nature (IUCN). We used spatial data on mammal AOH in Rondinini et al. (2011). We excluded species considered extinct and any other extant native and reintroduced species whose AOH maps did not fully intersect with the combined spatial data sets we used. In our analyses, we included 4460 species and excluded 1070 species, many of which had a portion of their range on islands and other features outside the extent of our combined spatial intersection layers.

Globally, more than 370 million people in more than 70 countries self‐identify as Indigenous (Garnett et al. 2018). We used a recently compiled global spatial data set on Indigenous Peoples’ lands located or delineated on the basis of open‐access published sources (Garnett et al. 2018) that, although incomplete, is the best currently available spatial layer at a global scale.

### Spatial Data on Human Pressure

Advances in remote sensing coupled with bottom‐up survey data have enabled the development of a spatially explicit, validated, high‐resolution global data set on human pressures (Venter et al. 2016). These data sets permit the quantification of the extent of intense pressures on individual species (Di Marco et al. 2018; Allan et al. 2019; O'Bryan et al. 2020). We used the most current human footprint map available (2013) (Williams et al. 2020), which contains a composite spatial index of key human pressures on natural ecosystems at a 1‐km^2^ resolution.

We used all 8 human‐pressure variables in the human footprint: built environments, population density, electrical infrastructure, crop lands, pasture lands, roads, railways, and navigable waterways. These pressures were scaled between 0 and 10 based on their estimated environmental impact and summed in 1‐km^2^ grid cells. Some pressures co‐occurred, whereas others were mutually exclusive, which resulted in a combined global scale of 0–50, where 0 had no detectable change and 50 was extreme urban conglomerates. We reclassified the human footprint map to a discrete index threshold of <3 because this threshold is considered the standard for evaluating the degree of low human pressure across ecosystems (Di Marco et al. 2018; Jones et al. 2018; O'Bryan et al. 2020). A threshold of approximately 3 is the level at which areas with low states of human pressure transition to human‐dominated activities, such as pastureland. Importantly, index values at or >3 reveal an increased extinction risk in mammals (Di Marco et al. 2018).

### Analyses

We combined the spatial data sets on Indigenous Peoples’ lands (mean individual size of 485.52 km^2^ [SD 34,348.43]) and low‐pressure lands (i.e., human footprint index <3) into a single spatial data layer based on overlap with the center of the pixel in a geographic information system raster calculator (ESRI ArcGIS, Redlands, California) at a 1‐km^2^ resolution (45.2% of Indigenous Peoples’ lands contain low‐pressure lands). We calculated the proportion of mammal species’ habitat in all Indigenous Peoples’ lands and in low‐pressure Indigenous Peoples’ lands by intersecting individual species’ AOH rasters with the combined spatial data set mentioned above with R statistical software (R Core Team 2017). Mammals were not included if their raster layer did not fully overlap with the intersection layer.

## Results

### Occurrence of Species in Indigenous Peoples’ Lands

**Figure 1:**
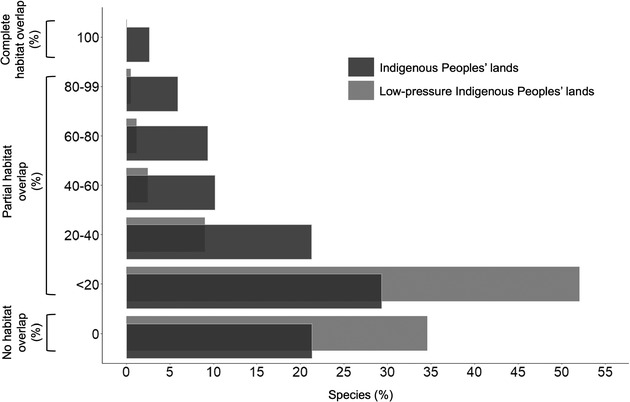
The percent area of terrestrial mammal habitat that overlaps mapped Indigenous Peoples’ lands (Garnett et al. 2018; dark gray bars) and low‐pressure Indigenous Peoples’ lands (i.e., < 3 on the human footprint index [light gray bars]).

**Figure 2:**
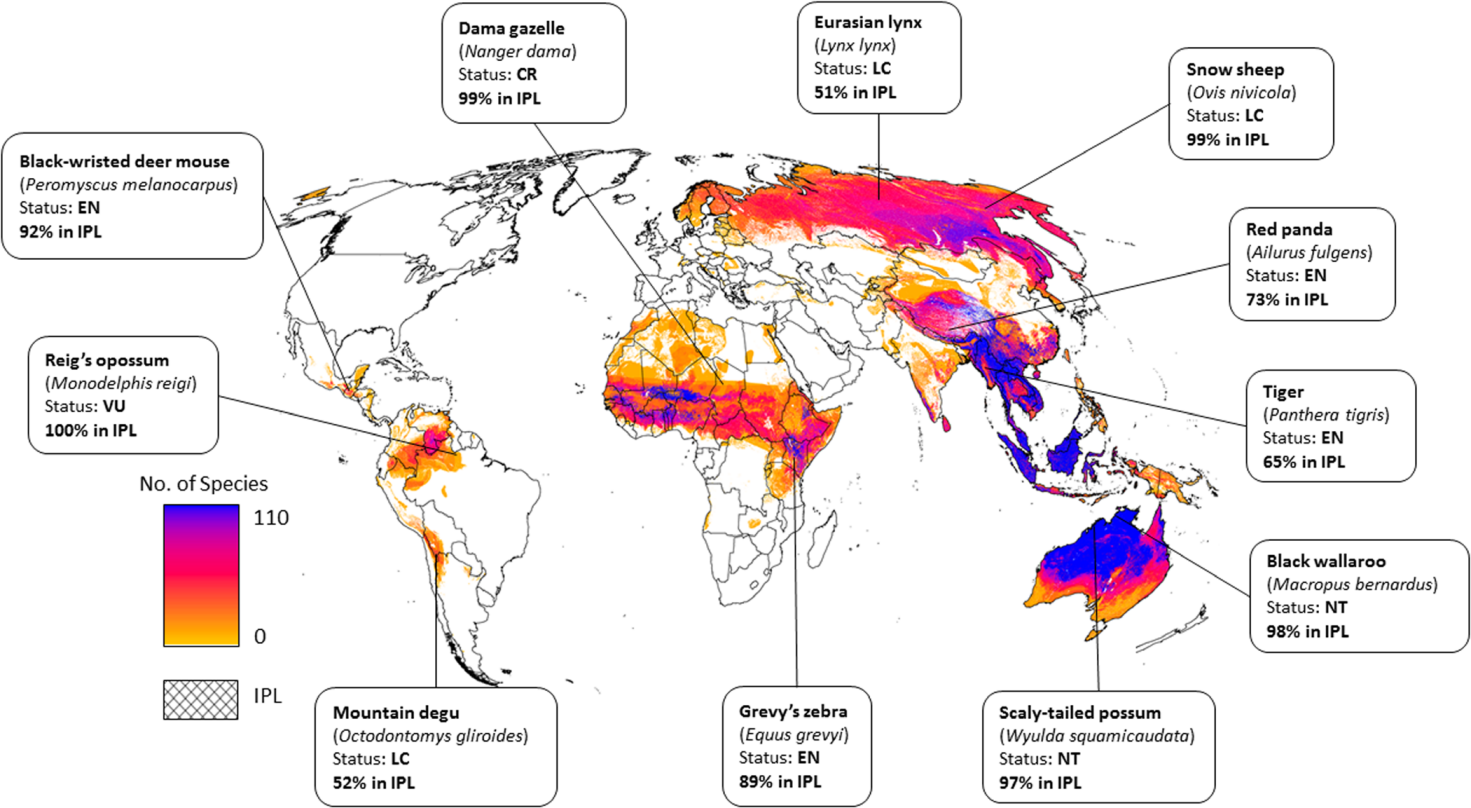
Number of species that have >50% of their habitat in mapped Indigenous Peoples’ lands (IPL) and locations of a subset of species (hatching, mapped Indigenous Peoples’ lands [Garnett et al. 2018]; LC, least concern; NT, near threatened; EN, endangered; CR, critically endangered).

We found that 2695 (60.4%) of all mammal species assessed had at least 10% of their habitat on Indigenous Peoples’ lands, and 1009 (22.6%) had >50% of their habitat in these lands (Fig. 1). Mammals in the order Scandentia (treeshrews of Southeast Asia) had the highest average percentage of their habitat overlapping with Indigenous Peoples’ lands (63.0% [SD 24.5]). For comparison, the orders Peramelemorphia (bandicoots and bilbies of Australia) had an overlap of 42.4% on average (SD 37.9), whereas Dasyuromorphia (carnivorous marsupials of Australia) and Perissodactyla (odd‐toed ungulates) had an average 40.9% (SD 30.5) and 39.7% (SD 32.0), respectively. Southeast Asia, northern Asia, Oceania, the grassland and semiarid regions of Africa, and northern South America had the highest number of species with >50% of their range on Indigenous Peoples’ lands (Fig. 2). For example, tigers (*Panthera tigris*) and red pandas (Ailurus fulgens) had 65% and 73% of their habitat in Indigenous Peoples’ lands, respectively (Fig. 2).

Of the 1002 mammal species assessed that were classified as threatened (i.e., vulnerable, endangered, or critically endangered [IUCN 2019]), 473 (47.2%) had at least 10% of their habitat on Indigenous Peoples’ lands; 255 (25.4%) species were vulnerable, 156 (15.6%) were endangered, and 62 (6.2%) were critically endangered. We also found that 261 (26.0%) of all threatened species had >50% of their ranges on these lands; 132 (13.2%) species were vulnerable, 95 (9.5%) were endangered, and 34 (3.4%) were critically endangered.

### Occurrence of Species in Low‐Pressure Indigenous Peoples’ Lands

Nearly 21 million km^2^ of Indigenous Peoples’ lands had low pressure (15.5% of terrestrial Earth, and 45.2% of all Indigenous Peoples’ lands [Appendix S1]). We found that 935 (21.0%) of species assessed had at least 10% of their habitat in these low‐pressure Indigenous Peoples’ lands; 118 (2.6%) had >50% of their habitat in these lands (Fig. 1). Mammals in the order Dasyuromorphia (carnivorous marsupials of Australia) had the highest average percentage of their habitat in these lands (23.7% [SD 24.2]). For comparison, the orders Pilosa (anteaters and sloths of the Americas) and Diprotodontia (noncarnivorous marsupials of Australia) had 19.0% (SD 11.1) and 15.6% (SD 21.3) of their habitat on Indigenous lands.

**Figure 3:**
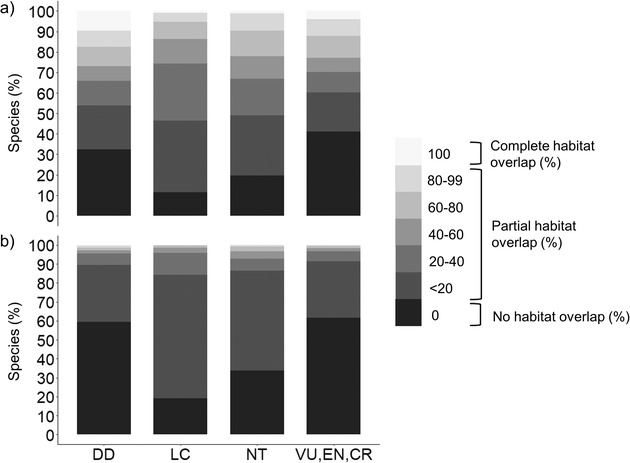
The percent area of terrestrial mammal habitat overlap with mapped Indigenous Peoples’ lands (Garnett et al. 2018) by International Union for Conservation of Nature Red List category (DD, data deficient; LC, least concern; NT, near threatened; VU, vulnerable; EN, endangered; CR, critically endangered) for (a) all Indigenous Peoples’ lands and (b) low‐pressure Indigenous Peoples’ lands (i.e., < 3 on the human footprint index).

Not surprisingly, the percentage of threatened species on low‐pressure Indigenous Peoples’ lands was considerably lower than that of threatened species across all Indigenous Peoples’ lands (Fig. 3). Of the threatened species assessed, 131 (13.1%) had at least 10% of their habitat in low‐pressure Indigenous Peoples’ lands. Eighty‐one (8.1%) of these species were vulnerable, 35 (3.5%) endangered, and 15 (1.5%) critically endangered. We also estimated that 25 (2.5%) of the threatened species assessed had >50% of their habitat in these lands. Of these, 19 (1.9%) were vulnerable, 5 (0.5%) were endangered, and 1 (0.1%) was critically endangered (Fig. 3b).

## Discussion

Indigenous Peoples’ lands cover at least 38 million km^2^ (28.3%) of Earth's land surface (Garnett et al. 2018) and include some of the highest quality forest lands worldwide (Fa et al. 2020). It follows that Indigenous Peoples are stewards of a substantial proportion of Earth's biodiversity. Although it has long been suspected that the proportion of biodiversity that occurs on Indigenous Peoples’ lands was likely to be high (Toledo 2013), our study is to the best of our knowledge the first to use robust, repeatable methods for determining this at the global scale. The numbers we derived are substantial. Globally, 60% of all mammals assessed and 47% of threatened mammals assessed had ≥10% of their habitat within Indigenous Peoples’ lands. For 23% of mammals, including 26% of threatened mammals, the proportion of their habitat was >50%, suggesting that Indigenous Peoples’ lands contain critical habitat for many assessed mammalian species.

Indigenous Peoples’ lands with low human pressure contained at least 10% habitat for 935 species. Such areas may serve as critical refugia from anthropogenic threats, especially for the 131 threatened species with at least 10% of their habitat on these lands, which require safeguarding from ongoing and future habitat loss and exploitation pressures. Our results showed that 57% of species that had some portion of their habitat on Indigenous Peoples’ lands may also be exposed to increased unsustainable human pressure on these lands (i.e., human footprint index ≥3), pointing to an even greater need for Indigenous‐led and collaborative conservation efforts. Pressure to exploit Indigenous Peoples’ lands and in some cases deny their rights to use and access these areas is alarmingly high all over the world (Fernández‐Llamazares et al. 2020; Scheidel et al. 2020).

Our results highlight future opportunities for improving understanding of species composition and open up important conservation agendas to build alliances that respect Indigenous rights and agendas. For example, the taxonomic group for which we had AOH data—mammals—is but a small fraction of the biodiversity found, and there is great opportunity for expanding this work to other taxonomic groups as AOH data become more accessible (Brooks et al. 2019). However, our results, based on best available globally consistent mammal data, may likely be true for other vertebrates (Leal et al. 2010), as well as plants, invertebrates, and other forms of biodiversity (but see Oberprieler et al. [2019]). Future work can also improve temporal overlap of species’ habitat layers with mapped Indigenous Peoples’ lands and human footprint data because our analysis was limited to spatial data across varying periods. For example, the AOH maps were published in 2011, the maps of Indigenous Peoples’ lands in 2017, and the human footprint data are from 2013. Temporal mismatch may be reduced as species AOH data become more widely available both spatially and temporally across taxonomic groups (Brooks et al. 2019).

The mapped Indigenous Peoples’ lands data we used were incomplete and may under‐ or overestimate coverage of Indigenous Peoples’ lands, depending on if and how groups self‐identify as Indigenous Peoples’ and how lands are defined (Garnett et al. 2018). Moreover, because stringent legislation often controls access to and activities on Indigenous Peoples’ lands, affecting the extent to which biodiversity is documented and mapped (dos Santos et al. 2015), it is very likely that survey efforts in these lands are incomplete (e.g., Bernard et al. 2011). Partnerships to help Indigenous Peoples fill knowledge gaps about significant and threatened species (including those that are culturally significant to local communities) will greatly improve understanding of the conservation status and population trends of these species and measures needed for their survival (Johnson et al. 2015; Garnett et al. 2018).

Myriad examples are available of how collaboration between Indigenous Peoples and researchers has refined knowledge of species ecological distribution ranges, baselines, and trends and opened up new understandings of biodiversity conservation that takes into account Indigenous rights, values, and aspirations (e.g., Ross et al. 2009; Mistry & Berardi 2016; Skroblin et al. 2019). However, such knowledge partnerships need to be negotiated and provide appropriate benefits to local Indigenous People (Robinson et al. 2016). The central message from our analysis is that Indigenous Peoples’ lands are vital to any policies and programs aiming to further global biodiversity conservation. This conclusion strongly aligns with that of the Intergovernmental Science‐Policy Platform on Biodiversity and Ecosystem Services (IPBES) (Diaz et al. 2019; IPBES 2019) and results of many other studies (e.g., Dinerstein et al. 2019; Reyes‐García et al. 2019).

Our results point to the fact that, regardless of what results from discussions through the CBD about species and ecosystem targets in the post‐2020 Global Biodiversity Framework, Indigenous Peoples will play a globally important role in the conservation of biodiversity into the future. Indigenous Peoples’ rights must be fully respected, including their full and effective participation in developing laws, policies, and programs that affect them. Although representatives of Indigenous Peoples are engaging in global environmental forums through frameworks such as IPBES, the Intergovernmental Panel on Climate Change, and the CBD, this often occurs in the face of substantial barriers to engagement related to scale, knowledge, and power (Brugnach et al. 2017). Greater recognition and support for the close relationships that Indigenous Peoples have with their lands and natural resources is, therefore, a pressing imperative from the perspective of both social equity and biodiversity conservation (Howitt 2018). Only through rights‐based, equitable, and respectful partnerships and other forms of dialogue and collaboration with Indigenous Peoples will it be possible to ensure the long‐term and equitable conservation of biodiversity.

## Supporting information

Additional information is available online in the Supporting Information section at the end of the online article. The authors are solely responsible for the content and functionality of these materials. Queries (other than absence of the material) should be directed to the corresponding author.
