# Supplementary material for: The importance of Indigenous Peoples’ lands for the conservation of terrestrial mammals
Source: Conserv Biol. 2020 Dec 30;35(3):1002–8. doi: 10.1111/cobi.13620 (PMC8247428; doi:10.1111/cobi.13620)
Supplement: Supplementary file 1 — Additional information is available online in the Supporting Information section at the end of the online article. The authors are solely responsible for the content and functionality of these materials. Queries (other than absence of the material) should be directed to the corresponding author. [file COBI-35-1002-s001.docx]

**Supplementary Material**

The importance of Indigenous Peoples’ lands for the conservation of terrestrial mammals

**This file contains: 1 Table**

| **Land Categories** | **Area (km^2^)** | **Proportion of Earth’s terrestrial surface** |
| --- | --- | --- |
| Total terrestrial surface | 134,154,306 | 1.0000 |
| Total Indigenous Peoples’ lands | 38,001,845 | 0.2833 |
| Pressure-free Indigenous Peoples’ lands | 20,825,442 | 0.1552 |
| Formally protected Indigenous Peoples Lands | 7,077,09 | 0.02666 |

**Table S1.** The area of each land category used for our study, and the proportion of each on Earth’s terrestrial surface.

Data availability

Species distribution data are available from (Rondinini et al. 2011) upon request. Data used for Indigenous Peoples’ land mapping are provided in Supplementary Information section of Garnett and colleagues (Garnett et al. 2018) and the derived maps are available upon request. The Human Footprint data are available from (Williams et al. 2020) upon request.

References

Garnett ST et al. 2018. A spatial overview of the global importance of Indigenous lands for conservation. Nature Sustainability **1**:369–374. Nature Publishing Group.

Rondinini C et al. 2011. Global habitat suitability models of terrestrial mammals. Philosophical Transactions of the Royal Society B: Biological Sciences **366**:2633–2641. Royal Society.

Williams BA et al. 2020. Change in Terrestrial Human Footprint Drives Continued Loss of Intact Ecosystems. SSRN Scholarly Paper ID 3600547. Social Science Research Network, Rochester, NY. Available from https://papers.ssrn.com/abstract=3600547 (accessed June 11, 2020).
